# Supplementary figures and images for: Ginkgo biloba extract increases neurite outgrowth and activates the Akt/mTOR pathway
Source: PLoS One. 2019 Dec 2;14(12):e0225761. doi: 10.1371/journal.pone.0225761 (PMC6886765; doi:10.1371/journal.pone.0225761)

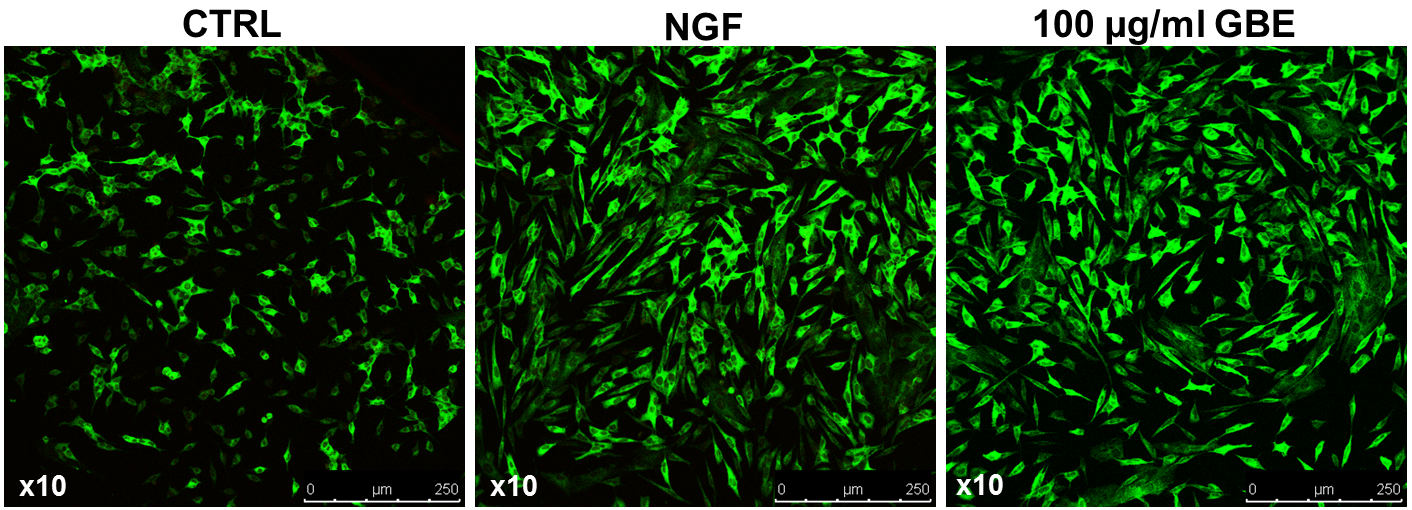

Supplement: S1 Fig — (TIF) [file pone.0225761.s001.tif]

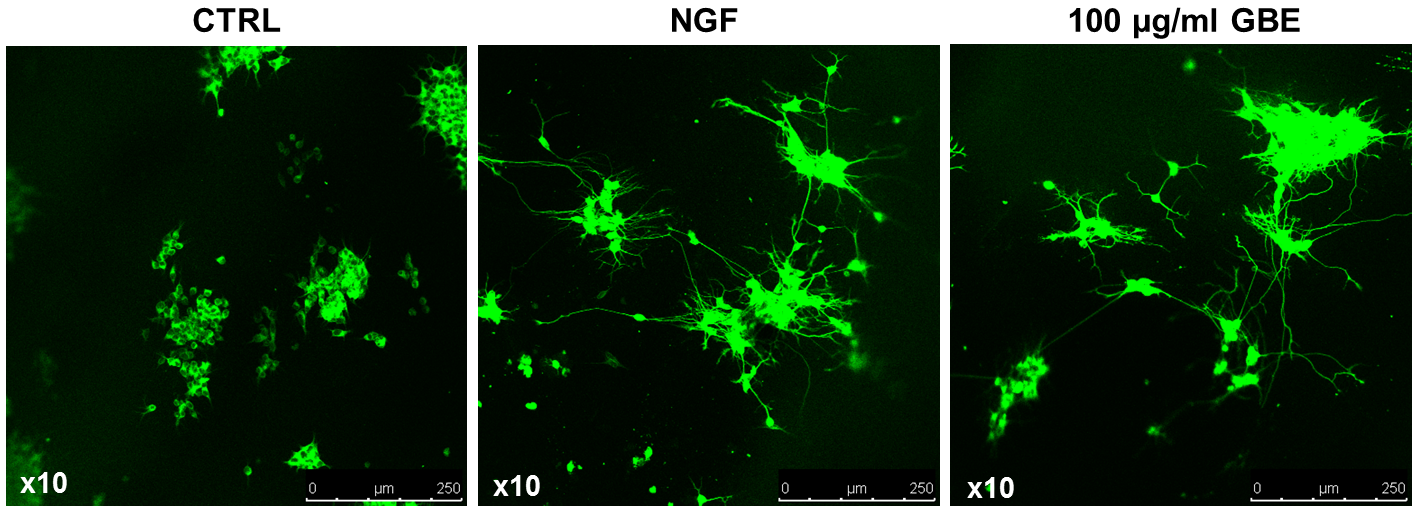

Supplement: S2 Fig — (TIF) [file pone.0225761.s002.tif]
